# Supplementary material for: Suppression of annexin A1 and its receptor reduces herpes simplex virus 1 lethality in mice
Source: PLoS Pathog. 2022 Aug 8;18(8):e1010692. doi: 10.1371/journal.ppat.1010692 (PMC9359538; doi:10.1371/journal.ppat.1010692)
Supplement: S1 File — (DOCX) [file ppat.1010692.s002.docx]

**Supporting materials and methods**

**Extraction of Anx-A1 on the cell surface and western blot analysis**

To extract Anx-A1 on the cell surface, the cell monolayers were washed with phosphate-buffered saline three times and treated with ethylenediaminetetraacetic acid (EDTA) at 1 mM supplemented with protease inhibitors (Sigma-Aldrich) at room temperature for 2 minutes (min). The proteins in the EDTA wash were concentrated by precipitation with 10% trichloroacetic acid for 1 h on ice and washed twice with cold acetone. The EDTA-treated cells and mouse tissues were lysed with buffer (10 mM Tris-HCl at pH 7.5, 150 mM NaCl, 5 mM EDTA, 5 mM NaN_3_, 10 mM sodium pyrophosphate, and 1% Triton X-100) supplemented with protease inhibitors. Mouse tissues were harvested, frozen, and homogenized in lysis buffer (10 mM Tris-HCl at pH 7.5, 150 mM NaCl, 5 mM EDTA, 5 mM NaN_3_, 10 mM sodium pyrophosphate, and 1% Triton X-100) supplemented with protease inhibitors. The lysates were subjected to western blotting with Abs against Anx-A1, α-tubulin (Santa Cruz Biotechnology), or β-actin (clone AC-15, Sigma-Aldrich) and HRP-conjugated secondary Abs.

**Immunofluorescence staining and flow cytometry**

Uninfected A549 cells were fixed and stained with phalloidin conjugated with tetramethylrhodamine (Thermo Fisher Scientific) before or after permeabilization with 1× PBS with 0.1% Triton X-100. To stain HSV-1 bound to the cell surface, A549 cells were mock-infected or infected with HSV-1 KOS (MOI = 10) labeled with DiD at 4°C for 1 h and incubated at 37°C for 5 min to enhance virus binding to the cell surface. Cells were stained with anti-Anx-A1 Ab at 4°C for 2 h before fixation and permeabilization. Nuclei were stained with Hoechst 33258. Paraffin sections of mouse tissues were stained with Abs against Anx-A1, mouse keratin K3 (clone AE5; Millipore), mouse NeuN (clone A60; Merck Millipore or clone D4G4O; Cell Signaling), or HSV-1 (Dako). Alexa 488- or Alexa 594-conjugated secondary Abs (Invitrogen) were used to visualize signals in fixed cells and sections under a fluorescence microscope. For FPR1 detection, cells were trypsinized and stained with an Ab against FPR1 (clone 350418, R&D Systems) conjugated with fluorescein for detection of FPR1 levels on the cell surface by FACSCalibur. Data were analyzed by FlowJo software.

**DiD-labeled HSV-1 virions**

Virions were labeled with the lipophilic tracer DiD and purified as previously described [1, 2]. Virus-containing medium was centrifuged at 2,020 × g for 10 min and 10,000 × g for 30 min at 4°C, and virus was labeled with 5 μM of DiD (Invitrogen) for 15 min and purified using a discontinuous sucrose gradient.

**Boc-Met-Leu-Phe (Boc-1) treatment**

To inhibit FPR1 binding to Anx-A1, cells were treated with Boc-1 (BACHEM) for 1 h, infected with HSV-1 (MOI = 1) for another h, washed, and harvested at 4°C to determine the number of virus binding on cells by plaque assay.

**LC-MS/MS mass spectrometric analysis**

The viral proteins interacting with Anx-A1 in lysates of mock-infected or KOS-infected A549 cells were immunoprecipitated using anti-Anx-A1 Ab and subjected to LC-MS/MS analysis (Genomics BioSci & Tech, Ltd.). Briefly, the immunoprecipitants were digested with trypsin, and the resulting peptides were analyzed by Thermo LTQ Orbitrap XL (Thermo Fisher Scientific Inc.) and the Mascot database (<http://www.matrixscience.com/>). The peptide sequences were blasted using the UniPortKB/Swiss-Prot database to determine protein identification.

**References**

1. Cheshenko N, Liu W, Satlin LM, Herold BC. Multiple receptor interactions trigger release of membrane and intracellular calcium stores critical for herpes simplex virus entry. Molecular biology of the cell. 2007;18(8):3119-30. Epub 2007/06/08. doi: 10.1091/mbc.e07-01-0062. PubMed PMID: 17553929; PubMed Central PMCID: PMCPMC1949381.

2. Handler CG, Eisenberg RJ, Cohen GH. Oligomeric structure of glycoproteins in herpes simplex virus type 1. Journal of virology. 1996;70(9):6067-70. Epub 1996/09/01. PubMed PMID: 8709230; PubMed Central PMCID: PMCPMC190628.
