# Supplementary figures and images for: Suppression of annexin A1 and its receptor reduces herpes simplex virus 1 lethality in mice
Source: PLoS Pathog. 2022 Aug 8;18(8):e1010692. doi: 10.1371/journal.ppat.1010692 (PMC9359538; doi:10.1371/journal.ppat.1010692)

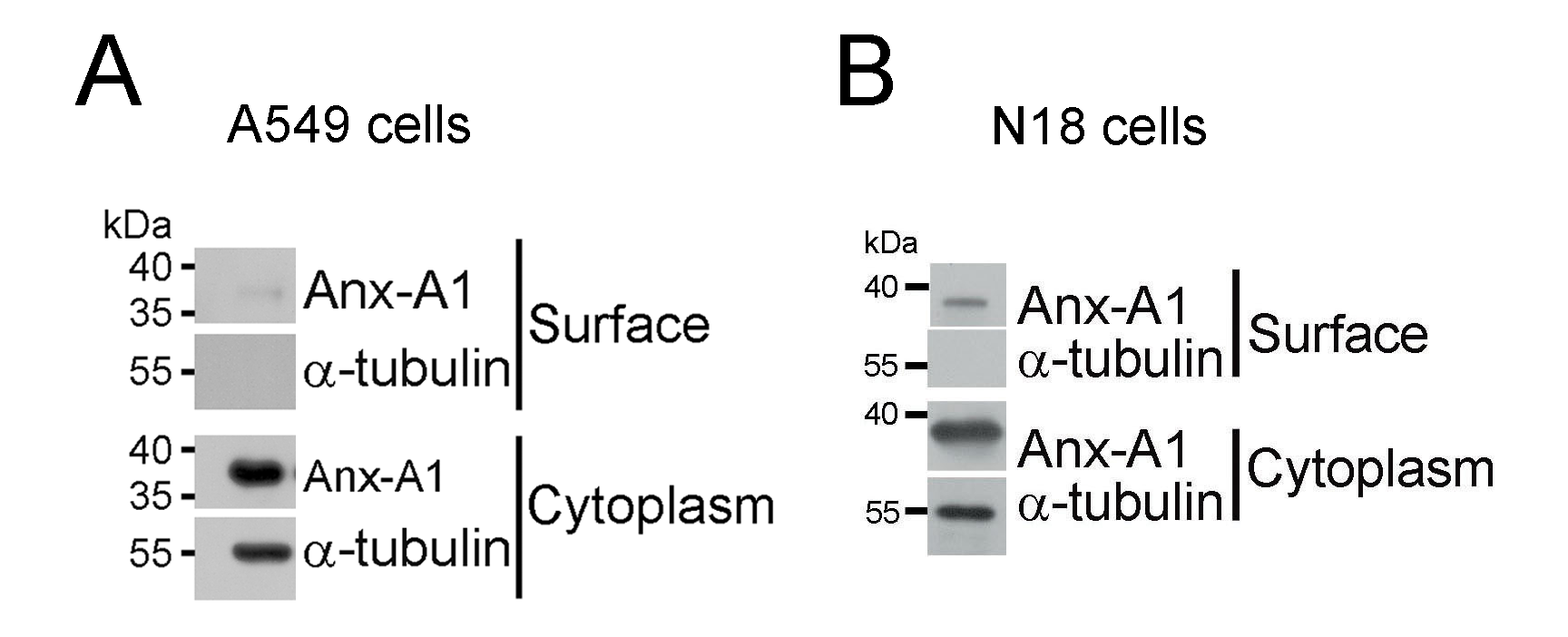

Supplement: S1 Fig — Representative western blots of Anx-A1 and α-tubulin on the surface and in the cytoplasm of mock-infected A549 cells (A) and N18 cells (B). (TIF) [file ppat.1010692.s003.tif]

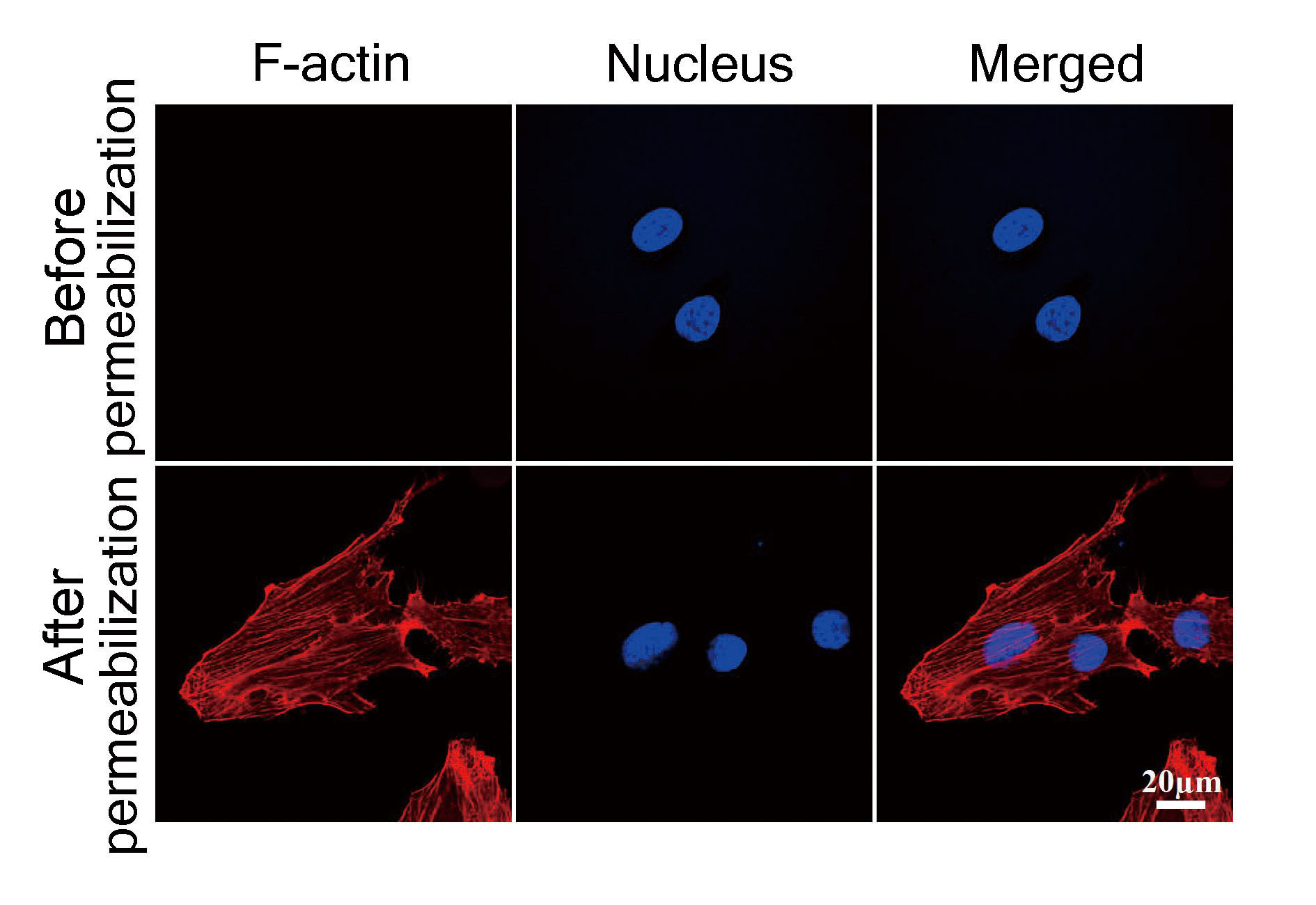

Supplement: S2 Fig — A549 cells were stained with phalloidin conjugated with tetramethylrhodamine to detect F-actin before and after permeabilization. Nuclei were counterstained with Hoechst. (TIF) [file ppat.1010692.s004.tif]

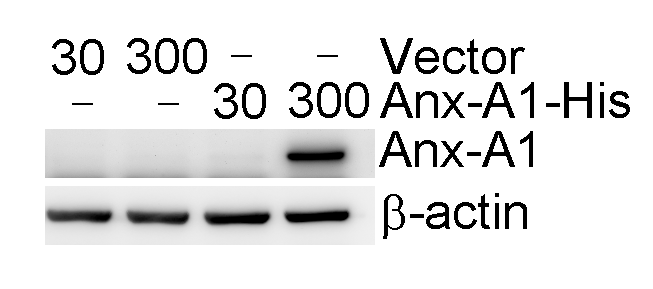

Supplement: S3 Fig — The representative western blots of indicated proteins in Anx-A1-/- MEFs transfected with 30 or 300 ng/culture of control vector (Vector) or the vector expressing Anx-A1 with His-tag (Anx-A1-His) for 24 hours are shown. The Anx-A1-/- MEFs transfected with 300 ng/culture plasmid DNA were subjected to HSV-1 infection shown in Fig 2B. (TIF) [file ppat.1010692.s005.tif]

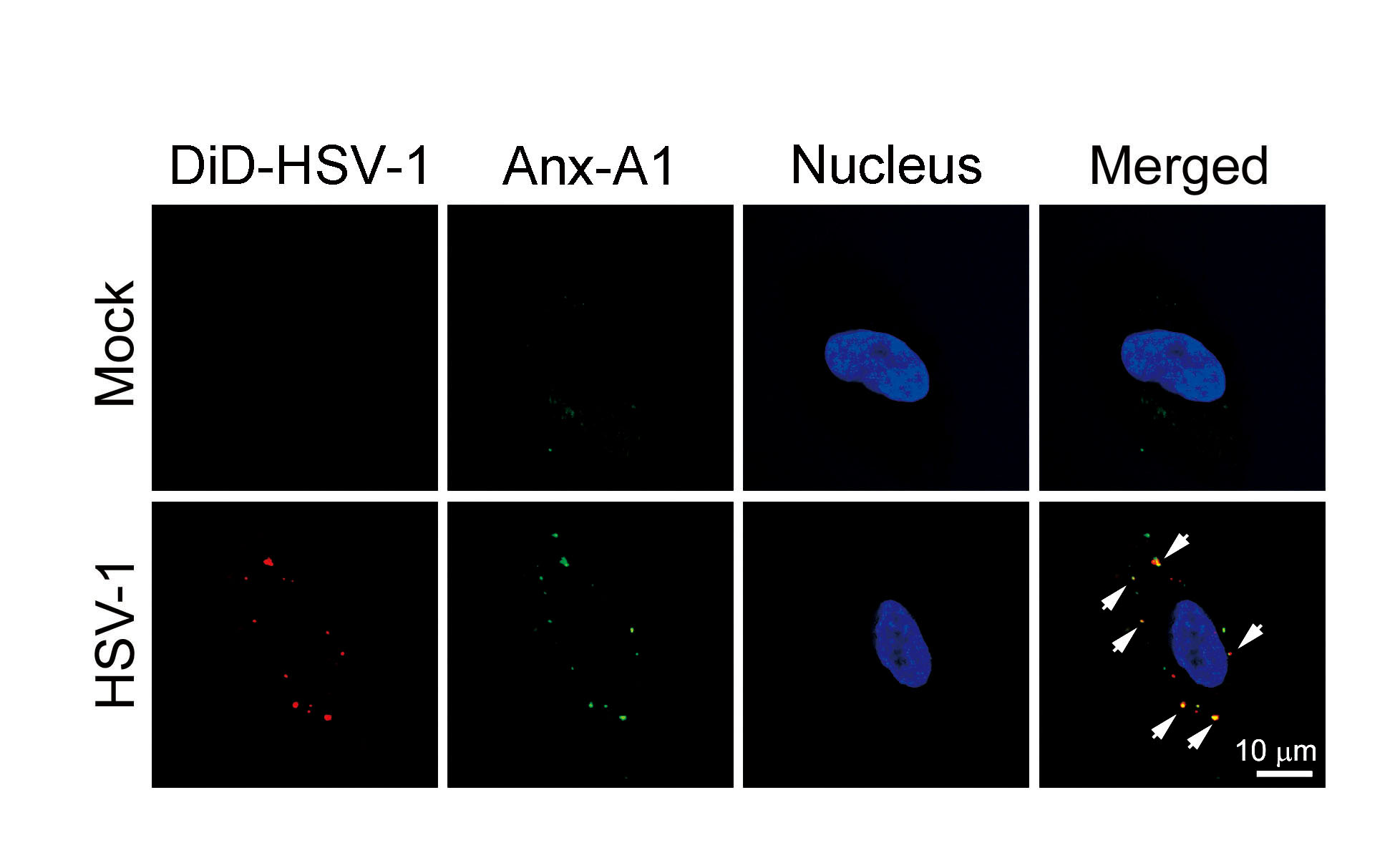

Supplement: S4 Fig — A549 cells were mock-infected or infected with HSV-1 KOS (MOI = 10) labeled with the lipophilic dye DiD at 4°C for 1 h before the cell culture temperature was shifted to 37°C for 5 min to enhance virus binding on the cell surface. Cell-surface Anx-A1 was stained with anti-Anx-A1 Ab, and nuclei were counterstained with Hoechst. Arrows indicate the colocalization of Anx-A1 with HSV-1. (TIF) [file ppat.1010692.s006.tif]

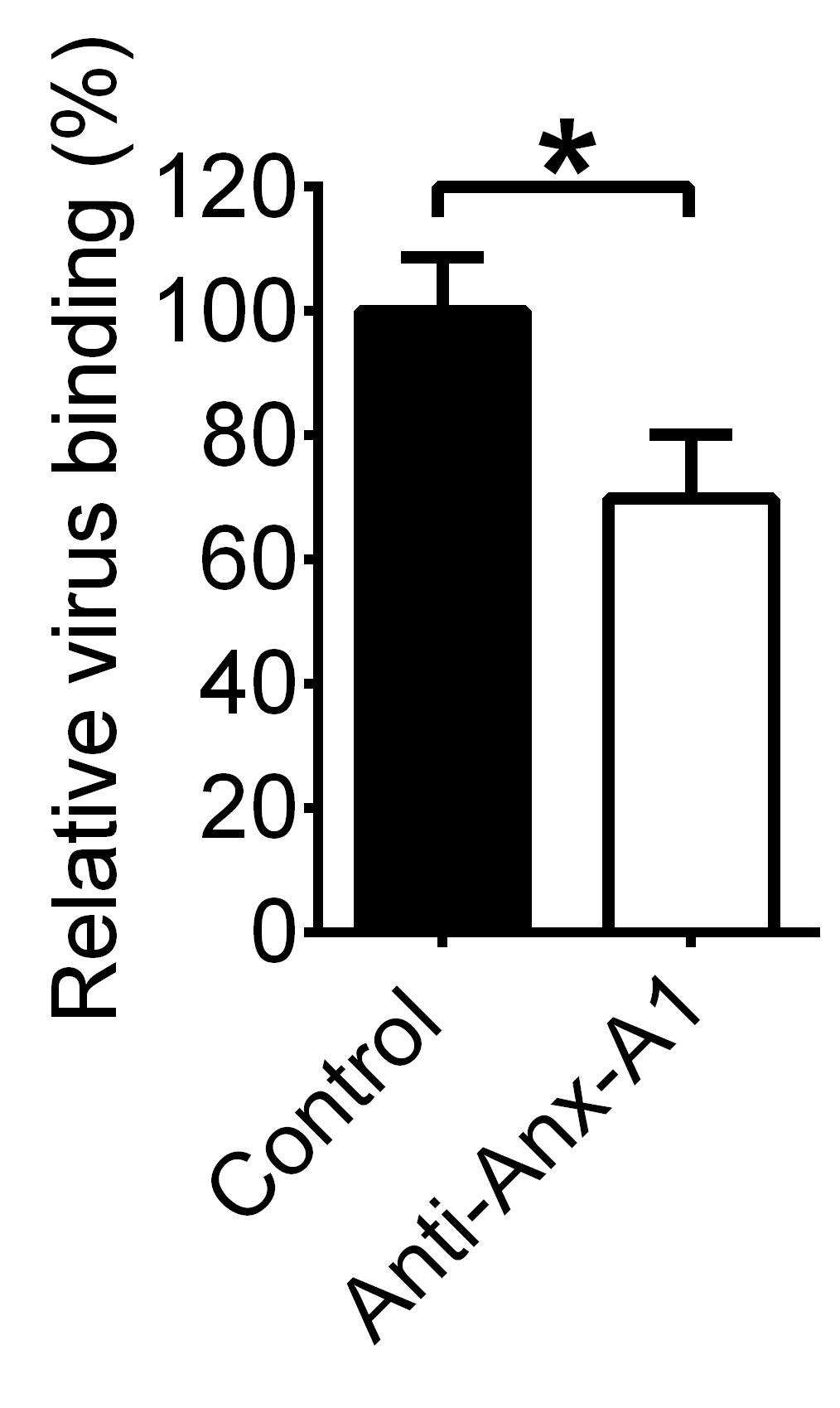

Supplement: S5 Fig — The level of virus binding to U-2 OS cells treated with control or anti-Anx-A1 serum and infected with HSV-1 (MOI = 1) are shown. The level of virus binding to control serum-treated cells was set as 100%. Data show the mean + SEM of >4 samples per group. *P < 0.05. (TIF) [file ppat.1010692.s007.tif]

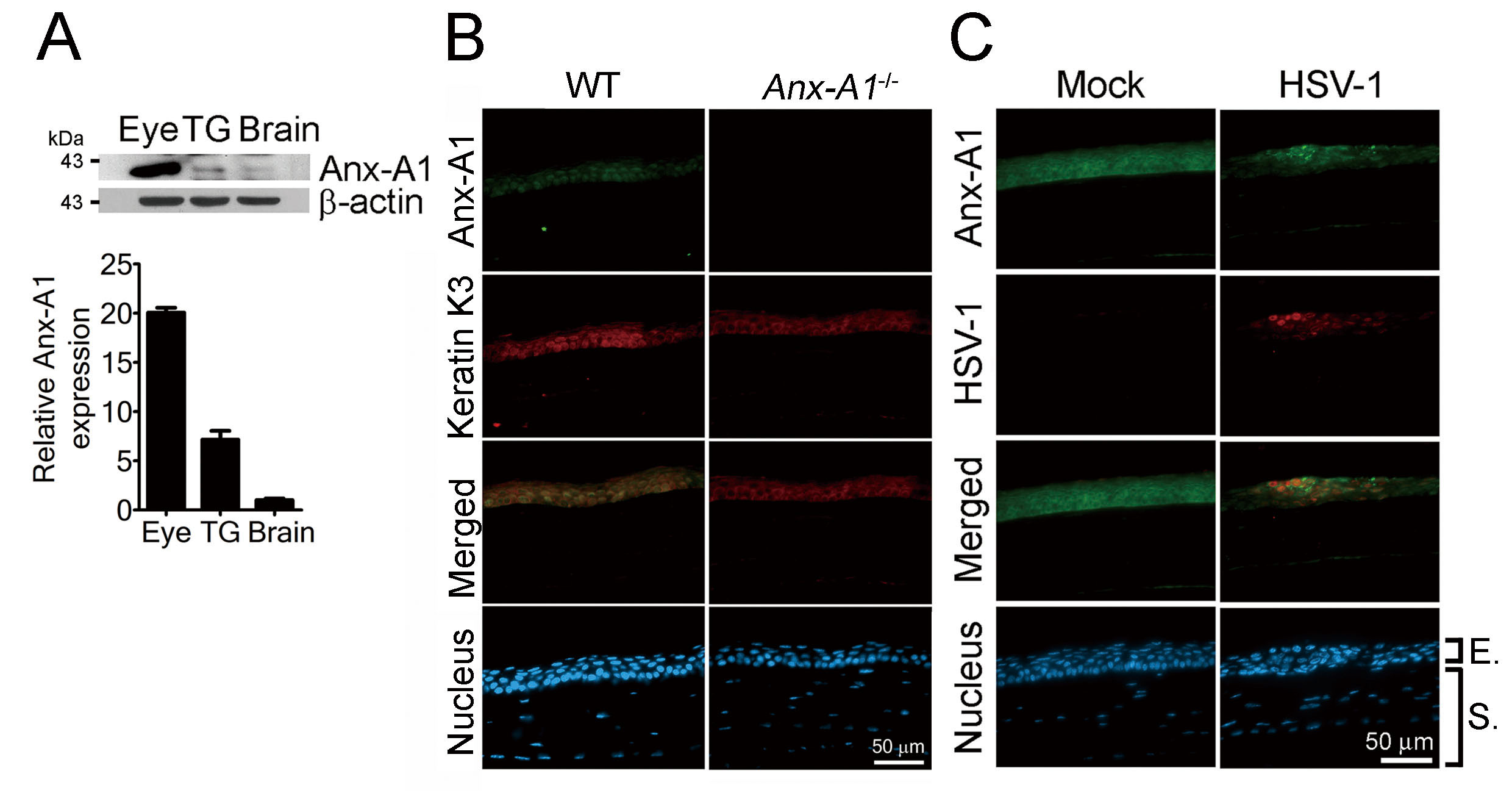

Supplement: S6 Fig — (A) Representative western blots of Anx-A1 and β-actin in the indicated tissues of mock-infected WT mice are shown in the top panel, and the quantitative result is shown in the bottom panel. The mean value of brain samples was set as 1. Data show the mean + SEM of 3 samples per group. (B) The eyes of uninfected WT and Anx-A1-/- mice were sectioned and stained with Abs against Anx-A1 or keratin K3. (C) The eyes of WT mice mock-infected or infected with HSV-1 294.1 for 1 day were sectioned and stained with Abs against Anx-A1 or HSV-1. E, epithelium. S, stroma. Nuclei were counterstained with Hoechst. Images are representative of at least 3 samples per group from 2 independent experiments. (TIF) [file ppat.1010692.s008.tif]

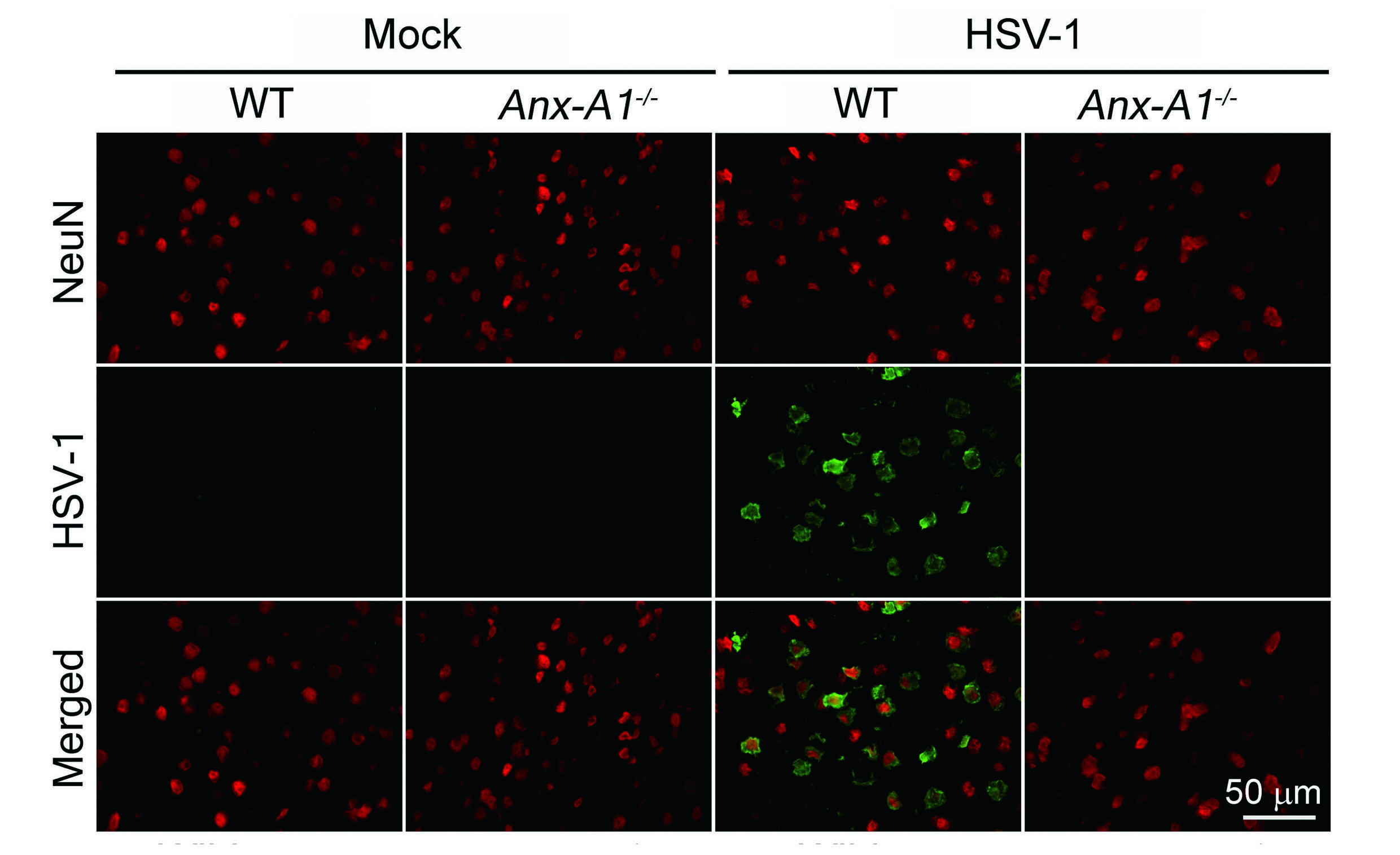

Supplement: S7 Fig — The representative images of mouse brains harvested at 7 dpi, sectioned, and stained with antibodies against NeuN or HSV-1 are shown. (TIF) [file ppat.1010692.s009.tif]

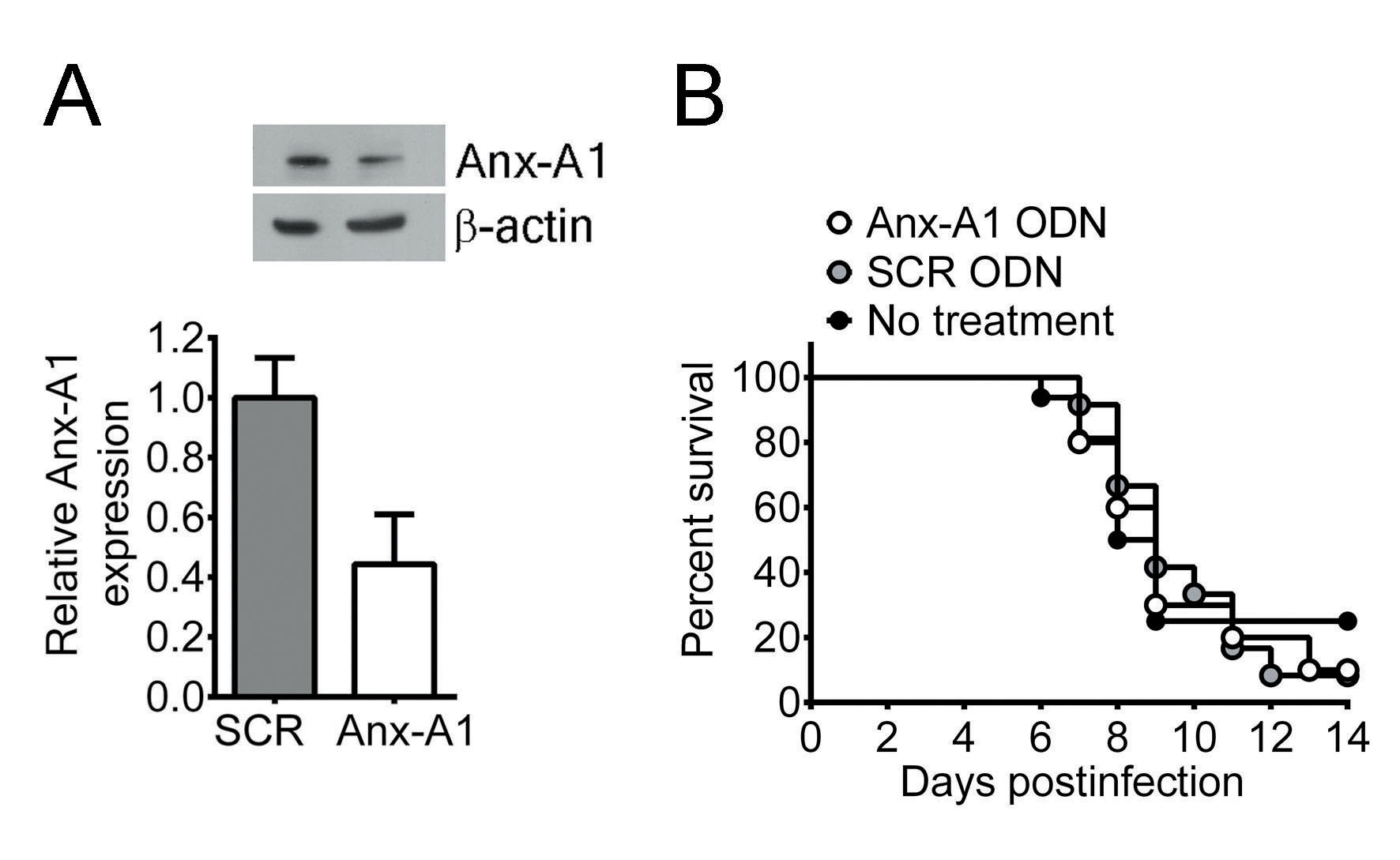

Supplement: S8 Fig — (A) Representative western blots (upper panel) and quantitative results (lower panel) of Anx-A1 expressed in the brains of mice treated with scramble (SCR) or Anx-A1 antisense ODN (shown in Fig 6A) and infected with virus for 7 days are shown. Data show the mean + SEM of 3 samples per group. The relative level of Anx-A1 in the brain of SCR ODN-treated mice was set as 1. (B) The survival rates of infected mice treated without ODN (No treatment; n = 16) or with reduced (one-third) amounts of scramble ODN (SCR; n = 12) or Anx-A1 ODN (n = 10) shown in Fig 6A, are shown. (TIF) [file ppat.1010692.s010.tif]

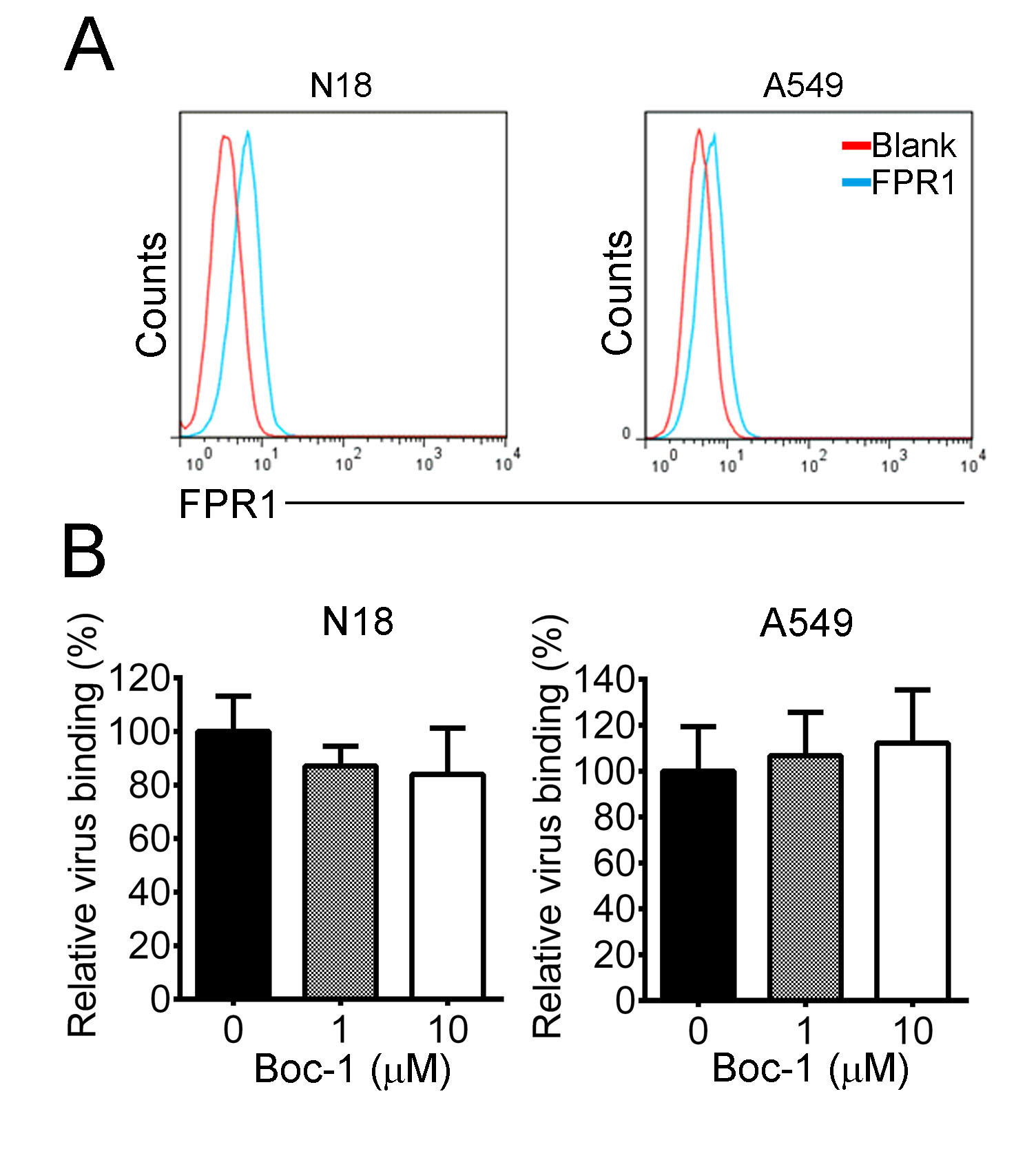

Supplement: S9 Fig — (A) Representative histograms of FPR1 detected on the surface of the indicated cells are shown. (B) The levels of virus binding to cells treated with the indicated concentrations of Boc-1 are shown. The level of virus binding on cells without Boc-1 treatment was set as 100%. Data show the mean + SEM of 6 samples per group. (TIF) [file ppat.1010692.s011.tif]
