# Supplementary material for: Suppression of annexin A1 and its receptor reduces herpes simplex virus 1 lethality in mice
Source: PLoS Pathog. 2022 Aug 8;18(8):e1010692. doi: 10.1371/journal.ppat.1010692 (PMC9359538; doi:10.1371/journal.ppat.1010692)
Supplement: Attachment — Submitted filename: 20220615 Responses to reviewers.docx [file ppat.1010692.s012.docx]

Responses to the review comments for

Manuscript ID: PPATHOGENS-D-21-02427 - [EMID:ea0fa315d07bef5d]

Title: Suppression of annexin A1 and its receptor reduces herpes simplex virus 1 lethality in mice

Reviewer #1

The authors provide evidence for their claim that HSV-1 glycoprotein gE binds to Anx-A1 on cell surface to promote infection of cells. Their in vivo results show that suppression of Anx-A1 reduces the mortality and viral loads in infected mice. Multiple in vitro assays as well as in vivo assays were performed to validate the claim. **The manuscript is well written and the results well described.**

(1) Anx-A1 is mostly cytosolic. Could it reduce cell-to-cell spread by neutralizing gE intracellularly? Similar things could happen when neighboring cells secrete the protein. This alternate possibility should be analyzed.

**Reply:** Anx-A1 is a cytosolic protein, and gE is a glycosylated protein on the plasma membrane, endoplasmic reticulum, and Golgi apparatus. As shown in **Fig 3D**, the cytoplasmic tail of gE is dispensable for Anx-A1 binding, and the extracellular domain of gE in the lumen of endoplasmic reticulum and Golgi apparatus is inaccessible for cytosolic Anx-A1 binding in cells. Hence, Anx-A1 is unlikely to neutralize gE intracellularly. Anx-A1 can be secreted or externalized from the cytosol. Our results showed that (the secreted or externalized) Anx-A1 was detected on the cell surface and HSV-1 envelops to promote HSV-1 attachment.

(2) Why did the authors choose not to study cell-to-cell spread when gE is a known player there? A simple cell mixing (infected to uninfected) assay can be done to demonstrate effect on spread of fluorescent virions.

**Reply:** We have addressed this issue in the Discussion (**lines 342-56**). HSV-1 gE is known to facilitate the cell-to-cell spread of HSV-1 [9, 21, 35], which is mediated by syncytium formation and by directing virus egress at the intercellular spaces [37]. Interestingly, Anx-A1 is shown to promote syncytium formation in reovirus- or measles virus-infected cells [38]. However, **the previous study using HSV-1 strain KOS [39] and our study using strains KOS and 294.1 found that both KOS and 294.1 fail to induce syncytium formation in MEFs and all cell lines tested** in our study. Further studies are needed to clarify the role of Anx-A1-gE interaction in promoting syncytium formation with the HSV-1 strain capable of inducing syncytium. **For directing HSV-1 egress and gE to the intercellular space, the gE cytoplasmic tail is required [40, 41]. However, the gE cytoplasmic tail is dispensable for Anx-A1 binding (Fig 3D), suggesting that Anx-A1-gE interaction does not direct HSV-1 egress at the intercellular space.** As Anx-A1 binds gE extracellular domain, we focused on Anx-A1 in promoting HSV-1 infection at the initial phase (attachment). We showed that blocking Anx-A1 or FPR2 decreases HSV-1 attachment to cells by 20 to 30% and ameliorates HSV-1-induced encephalitis in mice.

(3) Figs. 1/3, Results (and interpretations) are confusing. Anx-A1 expression goes up 1-2h later, by that time most virions should have been internalized. Several experts believe that virus entry happens within 5 min.

**Reply:** We agree with the Reviewer that most virions should have been internalized within minutes of infection, The other Review commented (#1) that the increased cell surface level of Anx-A1 was actually due to Anx-A1 on virions, which continued to bind to the cell surface after raising the temperature to 37°C. We cannot exclude this possibility, so in the Fig 1 of revised manuscript, we only show results that Anx-A1 can be detected on the surface of cells permissive for HSV-1 before infection, which can bind gE.

(4) Fig. 2. Results shown in this figure are very important. However, it is not clear whether a loading or input protein control was used.

**Reply:** We have modified **Fig 3** (original Fig 2) to show that equal amounts of total proteins (Input) were used for the co-immunoprecipitation assay, and β-actin was included as an input protein control.

(5) Fig. 4, results are confusing. Normally the presence of gE next to Anx-A1 on viral envelope should reduce the ability of gE to interact with Anx-A1 on the cell membrane.
    **Reply:** To address this issue, WT and *Anx-A1^-/-^* MEFs were infected with Anx-A1-positive or Anx-A1-negative HSV-1 (MOI = 1) for 1 h at 4°C, washed at 4°C, immediately stored at -80°C, and subjected to determine HSV-1 attachment on the cell surface. The results of **Fig 4D** showed that absence of Anx-A1 either on the cell surface or on viral envelopes abolished Anx-A1-enhanced HSV-1 attachment to MEFs. We have added **Fig 9** and sentences in the Discussion section (**lines 305-19**) to explain that the Anx-A1 (next to gE) on viral envelopes promotes HSV-1 attachment to cells via the Anx-A1 receptor (FPR2) and forms homodimers on the cell surface to promote membrane aggregation (the aggregation of cell membrane and viral envelops). Moreover, gE next to Anx-A1 on viral envelopes can still bind to other ligands on the cell surface.
(6) Fig. 5, Could the results shown here be due to stronger compensatory mechanisms that takeover the normal functions of Anx-A1?

**Reply:** As demonstrated in **Fig 2A** and **2B**, absence of Anx-A1 reduced HSV-1 replication in MEFs, and ectopic Anx-A1 expression in *Anx-A1^-/-^* MEFs restored HSV-1 replication. Additionally, absence of Anx-A1 or transient knockdown of Anx-A1 via specific ODN decreased tissue viral titers and mortality of infected mice (**Fig 5** and **6**, respectively). These results indicate that Anx-A1, but not other compensatory functions of Anx-A1, regulates HSV-1 replication.

Reviewer #2: Wang and colleagues provide evidence that the cell protein annexin A1 (Anx-A1) enhances attachment of HSV-1 to cells via an interaction with gE. Furthermore, that blocking this interaction reduces the lethality of the virus for mice after infection via the cornea. The experiments with mice consist of comparing wildtype and Anx-A1-negative mice, of treating mice with an oligonucleotide that reduces Anx-A1 expression, and of treating mice with a peptide that blocks binding of the soluble Anx-A1 to its cell surface receptor FPR2. In all cases, the data support a role for Anx-A1. The results of experiments with tissue culture are less straightforward, in part because some of the methods used are not described adequately. Details are below. **There is novelty and potential significance to the study, and the Discussion is written with appropriate references to prior work.** Comments on the execution and interpretation of the experiments using tissue culture systems are as follows.

(1) In the first experiment, cells were incubated with virus at an MOI of 5 for 1 h at 4C, then

incubated at 37C for various times before measurement of cell surface Anx-A1. There was an initial increase, but then the level went back down. The conclusion is that infection raised the level of Anx-A1 on the cell surface, and that UV-inactivated virus had a smaller effect. However, in a later experiment it was shown that Anx-A1 is actually present on virions, which suggests that the increased cell surface level of Anx-A1 was actually due to Anx-A1 on virions. This would be consistent with an initial increase, followed by a decrease, because there is no indication that the inoculum was removed after the 4C step. More virus could have bound after raising the temperature, followed by entry into cells resulting in a later decrease of virions at the cell surface. The UV virus was only tested after 1 h at 37C, at which point the level of Anx-A1 is not greatly different from that seen without the UV treatment.
**Reply:** Our previous result was obtained without removing the inoculum after 4°C step. We took the suggestion of Reviewer to redo the experiment and remove the inoculum after 4°C step and found that after 1 h at 37°C, HSV-1 infection failed to significantly increase the Anx-A1 level on the cell surface. In the Fig 1 of our revised manuscript, we only show the results that Anx-A1 can be detected on the surface of cells permissive for HSV-1 before infection, which can bind to gE.

(2) A virus binding assay is described:
Cells were “infected with HSV-1 for 1 h, washed, and harvested at 4C to determine the number of virus binding to cells by plaque assay.”
What is the significance of the plaque assay? This is not usually the way that virus binding is measured, and it is entirely unclear if the procedure is actually measuring binding or a combination of binding and a later step or steps.

**Reply:** We used the protocol (plaque assay) described previously [20] to assess virus binding. In the revised manuscript, we have added one more assay, real-time PCR to assess virus binding. Briefly, cells were infected with HSV-1 for 1 h at 4°C (a temperature that allows only virus binding to cells, but not a later step or steps of virus infection), washed with phosphate-buffered saline three times at 4°C, immediately stored at -80°C, and subjected to determine the numbers of virus binding via plaque assay (**Fig 2C** and **7C**) or via real-time PCR (**Fig 2D** and **7D**). The results **Fig 2C, 2D, 7C,** and **7D** show that the levels of HSV-1 attachment on MEFs in the absence of Anx-A1 or on N18 cells with WRW4 treatment were reduced about 30% regardless of the detection methods (plaque or PCR assay).

(3) In Figs 1C and 1D, immunofluorescence is used to detect increased Anx-A1 at the cell surface after infection. Quantitation shows an approximately 2-fold overall increase. The number of positive cells increases from 32% to 78%. This is sufficient to account for the overall 2-fold increase, so it is difficult to accept the report that fluorescence intensity also increased, i.e., that positive cells became more positive. The images do not support this. And again, is the overall increase the result of Anx-A1 being brought to cells on virions?

**Reply:** As the reply in the **Comment 1**, in the **revised Fig 1** (orginal Fig 1C), we have deleted the results, which show increased Anx-A1 at the cell surface after infection.

(4) In Fig 2A, a virus overlay protein blot assay is used to demonstrate that virions bind to Anx-A1. Details of sample preparation are not given, but presumably this is a denaturing gel, in which case virions are binding to denatured Anx-A1, which is an interesting and surprising observation.

**Reply:** A denaturing gel was used, and we have listed this information in the Materials and Methods section (**line 462**).

(5) An interaction between gE and Anx-A1 is demonstrated, and said to be independent of other virus proteins (Fig. 2C). Nevertheless, quantitation of the gel would be useful, comparing the fraction of total Anx-A1 that is co-IP’d with gE; is it the same when only gE is present versus when all virus glycoproteins are present?

**Reply:** We quantified **Fig 3C** (original Fig 2C) via the Image J software and found that the ratio of Anx-A1/gE (since we precipitated gE to detect Anx-A1 in immunoprecipitant) is 0.51 in HSV-1-infected cells (expressing all glycoproteins) and 0.44 in AdgE-infected cells (expressing only gE). The results support our conclusion that gE-Anx-A1 interaction does not require other HSV-1 proteins. We have added the ratio data in **lines 169-70**.

(6) Anx-A1 interacts with the extracellular domain of gE. The experiment to demonstrate that it does not interact with the cytoplasmic domain (Fig. 2D) suffers from the problem that the hydrophobic transmembrane region is also included in the construct. This would likely result in aggregation of the cytoplasmic domain, preventing any meaningful conclusion.

**Reply:** In the experiment, we also constructed gE without its cytoplasmic tail, but with the hydrophobic transmembrane region, and found that Anx-A1 bound gE lacking the cytoplasmic tail but with the hydrophobic transmembrane region (**Fig 3D**) (original Fig 2D). The results still support our conclusion that gE cytoplasmic tail is dispensable for Anx-A1 binding.

(7) Figs. 3A and 3C are concerned with the effect of anti-Anx-A1 serum on binding of virus to cells. The effect is measured via plaque assay. As with comment (2), this is unclear. How does a plaque assay measure virus binding? Also, how can antibody binding to the Anx-A1 on virions be eliminated as a major contributor to the result?
**Reply:** As the reply in the **Comment 2**, absence of Anx-A1 reduces 30% HSV-1 binding on cell surface regardless of detection methods. Results of **revised** **Fig 4D** shows that Anx-A1 on both cell surface and viral envelopes is required to enhance HSV-1 attachment. In the Anx-A1 neutralization assay, cells were treated with Anx-A1 antiserum before and during HSV-1 infection. Hence, Anx-A1 antiserum neutralizes Anx-A1 on the cell surface and viral envelopes to interfere with HSV-1 attachment. To clarify this issue, we modified the manuscript (in **lines 204-8**) and put the neutralization results (original Fig 3A and 3C) in **Fig 4F** and **4H**.

(8) Yields of virus are measured in Figs 3B and 3D. Time points are 24 h and 36 h. A greater effect of anti-Anx-A1 antibody is seen at 36 h. The problem with this protocol is that by 36 h, there will have been two cycles of infection. Therefore, the effect of the antibody on virus spread from cell to cell is also a factor. Curiously, in the next experiments, with Anx-A1-negative cells, the results suggest that spread is NOT affected. To eliminate this complication, it would be preferable to use a higher MOI for the initial infection in all of these experiments, and to measure the virus yield at no later than 24 h.

**Reply:** The **Fig 4G** (original Fig 3D) showed the viral yields of MEFs infected with HSV-1 (MOI = 1) and treated with control or anti-Anx-A1 serum at 24 hpi. We have listed the MOI and time point in the Result section (**lines 205-6**). The original Fig 3B results of N18 cells were performed at the MOI of 1 and showed that the mean viral yield in cells treated with Anx-A1 antiserum (11.0 ± 2.0) × 10^5^ PFU/ml) was significantly lower than that in cells treated with control serum (29.0 ± 3.7) × 10^5^ PFU/ml) at 36 hpi. Similar results were observed at 24 hpi showing that the mean viral yield was reduced three-fold in N18 cells treated with anti-Anx-A1 serum (2.1 ± 0.5) × 10^5^ PFU/ml) when compared with control serum (7.9 ± 4.0) × 10^5^ PFU/ml). To demonstrate that the number of HSV-1-infected cells is reduced by blocking extracellular Anx-A1 and strength our conclusion, N18 cells were treated without or with different dilutions of control or anti-Anx-A1 serum and infected with HSV-1 strain KOS-GFP (MOI = 10). The GFP-positive cells (HSV-1-infected cells) were analyzed at 17 hpi. Results of **revised Fig 4I** shows that neutralizing Anx-A1 via antiserum decreased the number of HSV-1-infected cells by 20 to 40% when compared with control serum.

(9) There is an experiment to investigate a role for Anx-A1 in virus entry, i.e., a post-binding step, but the experimental protocol is unusual. Virus was incubated with cells for 1 h at room temperature, followed immediately by a low pH treatment to inactivate virions still on the cell surface, and then incubation at 37C to allow plaque formation. The assay therefore measures a combination of virus binding and entry into cells at room temperature, which is not very informative.
The conventional way to assess entry is to begin with an initial virus attachment step at 4C. Next to raise the temperature to 37C to allow virus entry, with low pH treatment at various times after temperature increase. This allows the rate of virus entry to be determined.
**Reply:** As we showed that Anx-A1 facilitates HSV-1 attachment, it would be impossible to exclude the effect of Anx-A1 on HSV-1 binding in entry assay regardless of the temperature used in the protocol. The plaque formation assay in **Fig 2E** (original Fig 3E) performed with the method described above in the Reviewer comment (highlighted with underline) is designated to determine the importance of Anx-A1-mediated HSV-1 binding in subsequent infection steps. Results of **Fig 2C** and **2D (**obtained by plaque assay and real-time PCR, respectively) reveal that absence of Anx-A1 reduced HSV-1 attachment, and results of **Fig 2E** demonstrate that absence of Anx-A1 reduced the number of plaques formed on MEFs (the number of infected cells) by 30%, showing that Anx-A1 mainly facilitates HSV-1 attachment. We have clarified this point in **lines 131-42**.

(10) A virus lacking gE is used in Fig 3I. Do the authors have an explanation for why the level of binding (if that is what is being measured) when gE is absent but Anx-A1 is present is only half that of when gE is present but Anx-A1 is absent?

**Reply:** As the reply in the **Comment 2**, absence of Anx-A1 reduces 30% HSV-1 binding on the cell surface regardless of the detection methods. **Fig 4E** (original Fig 3I) shows that cell surface Anx-A1 enhances HSV-1 binding (by comparing the strain F binding to WT and *Anx-A1^-/-^* MEFs) and absence of gE abolished Anx-A1-mediated HSV-1 binding (by comparing strains F and F-gEβ binding to WT MEFs). The binding of strain F-gEβ to WT MEFs (42%), when gE is absent but Anx-A1 is present, was less than that of strain F to *Anx-A1^-/-^* MEFs (71%), when gE is present but Anx-A1 is absent, suggesting that gE might bind other cellular proteins in addition to Anx-A1. To address the question of Reviewer, we have discussed this issue in **lines 324-6**.

(11) Fig 4 shows that Anx-A1 is present on virions, as mentioned earlier. The statement is also made that Anx-A1 is “adjacent to gE”. This statement cannot be justified without comparing the proximity of Anx-A1 to other virus glycoproteins, which was not done.

**Reply:** As demonstrated in S1 Table and Fig 3A and 3B, the results show that only gE, but not other viral glycoproteins, binds Anx-A1. We, therefore, used immunogold electron microscopy to further confirm that Anx-A1 co-localized with gE on HSV-1 virions. To be more precise, we revised the sentence from “Anx-A1 was detected adjacent to gE” to “Anx-A1 was co-localized with gE” on the HSV-1 envelopes (in **lines 188-9**).

(12) For Figs 4D-4F, with cells lacking Anx-A1, the level of binding is said to be “slightly higher” for virus containing Anx-A1 than for virus lacking Anx-A1 at an MOI of 1, and with a more pronounced difference at lower MOIs. It is difficult to appreciate how this effect could be influenced by the MOI. Is it caused by the assay measuring something other than, or additional to, binding? Is it influenced by the unavoidable presence of non-infectious virions in the inoculum?
**Reply:** Our explanation for the original Fig 4D-4F is that HSV-1 (ligand) binding kinetics to its receptor(s) is not linear. At the low MOIs of HSV-1, the enhancing effect of Anx-A1was more prominent. We removed these results in our revised manuscript to focus on how Anx-A1-gE interaction affects HSV-1 attachment to cells and used the MOI of 1 for all binding assays. The **revised Fig 4D** is to determine the importance of Anx-A1 on the cell surface and viral envelopes. WT and *Anx-A1^-/-^* MEFs were infected with Anx-A1-positive or -negative HSV-1 (MOI = 1) to access virus attachment, and the results show that Anx-A1 on both cell surface and viral envelopes is required for HSV-1 attachment (in **lines 194-6**).

(13) In Fig 7, a peptide that blocks Anx-A1 binding to its cell surface receptor FPR2 reduces virus binding to cells. There are two negative controls that would strengthen the conclusion. One is to show that the peptide has no effect on virus binding to Anx-A1-negative cells. The other is to show that the peptide has no effect on gE-negative virus binding to Anx-A1-positive cells.
**Reply:** We have added the results of two negative controls requested by the Reviewer. The peptide WRW4 (that blocks Anx-A1 binding to its cell surface receptor FPR2) has no effect on (wild-type HSV-1) virus binding to Anx-A1-negative cells (**Fig 7C and 7D)** and on gE-negative virus (F-gEβ) binding to Anx-A1-positive (N18) cells (**Fig 7E)**.

Reviewer #2: Key modifications/new experiments:

(1) Experimental protocols must be changed so that the relevance of Anx-A1 to individual aspects of virus attachment, entry, and virus yield is measured, rather than a combination of them. Binding should be measured by an assay that does not allow for subsequent entry, and not by plaque assay. Entry, or more informatively, rate of entry should be measured by an assay that begins with attachment under conditions where entry cannot occur, and then proceeds to different lengths of time at 37C such that entry can occur. Virus yield should be measured up to, but not exceeding, 24 h post-infection, and with an appropriately high MOI, such that only one cycle of replication is possible. This avoids the complication of effects on virus spread, in addition to attachment and entry.

**Reply:** As the reply in the **Comment 2,** binding was measured at 4°C (a temperature that allows only virus binding to cells, but not a later step or steps of virus infection) by plaque assay as described previously [20]. In the revised manuscript, we have added one more assay, real-time PCR to assess virus binding. Similar results were obtained by both methods. We performed plaque formation assay on MEFs monolayers to confirm the importance of Anx-A1-enhanced virus binding in subsequent infection steps, such as entry, as we responded in the **Comment 9**. For viral yield results (**revised** **Fig 4G and 4I**), we performed studies at high MOIs and within 24 h as the Reviwer suggested and responded in the **Comment 8**.

(2) The effect of Anx-A1 on virions must be addressed. If this is the source of the increase in cell surface Anx-A1 after virus binding/infection, then one conclusion of the manuscript is invalidated. This conclusion is that infection results in exit of intracellular Anx-A1 from cells, such that it is then available to bind to the cell surface.
**Reply:** We have addressed this issue in the **Comment 1**.

(3) Negative controls are needed for experiments with FPR2. Does the peptide have an effect on virus binding to Anx-A1-negative cells, and does it have an effect on gE-negative virus binding to Anx-A1-positive cells? If it does, then a different interpretation of the results already in the manuscript would be needed.
**Reply:** We have included the negative controls suggested by the Reviewer in our revised manuscript and addressed the issue in the **Comment 13**.
